# Mediating roles of systemic immune-inflammation index and body mass index in the association between dietary inflammatory index and cardiovascular disease in diabetes

**DOI:** 10.1097/MD.0000000000050622

**Published:** 2026-09-11

**Authors:** Shaofang Tang, Hongtao Li, Zixun Wang, Baoping Wang, Zhiqing Li, Yin Li, Chenlin Dai

**Affiliations:** a Department of Endocrinology and Metabolism, Tianjin Medical University General Hospital, Tianjin, China; b The Second Internal Medicine, Corps Hospital of Tianjin Armed Police Force, Tianjin, China; c Tianjin Key Laboratory of Retinal Functions and Diseases, Tianjin Branch of National Clinical Research Center for Ocular Disease, Eye Institute and School of Optometry, Tianjin Medical University Eye Hospital, Tianjin, China; d Medical School, Tianjin University, Tianjin, China.

**Keywords:** cardiovascular disease (CVD), diabetes mellitus (DM), dietary inflammatory index (DII), NHANES, systemic immune-inflammation index (SII)

## Abstract

This study aimed to analyze the dietary inflammatory index (DII) and its impact on cardiovascular disease (CVD) in individuals with diabetes and to explore the role of the systemic immune-inflammation index (SII) and body mass index (BMI) in the relationship between DII and CVD. Data were obtained from the National Health and Nutrition Examination Survey 2009 to 2023. A generalized linear model was used to examine the influence of DII on CVD among individuals with diabetes. The mediation effect model was employed to examine the relationship between DII, SII, BMI, and CVD among individuals with diabetes. Among 5068 participants, 1243 (24.53%) had diabetes mellitus (DM) with CVD. DII was a risk factor for CVD among individuals with diabetes (odds ratio = 1.140, 95% confidence interval [CI]: 1.091–1.192, *P* < .001). BMI partially mediated the relationship between DII and CVD among adults with DM, with an effect coefficient of 0.005 (95% CI: 0.001–0.010). SII partially mediated the relationship between DII and CVD among adults with DM, with an effect coefficient of 0.008 (95% CI: 0.001–0.016). Among adults with DM, we should pay attention to monitoring the level of DII and reducing BMI, which may help reduce the risk of comorbid CVD. Furthermore, the assessment and identification of CVD risk among diabetic patients with a high DII using the SII is crucial.

## 1. Introduction

Diabetes mellitus (DM) is a chronic noncommunicable disease with an increasing prevalence, posing a major public health challenge worldwide. According to statistics, approximately 463 million people were affected by DM globally in 2019, with projections suggesting that this number will rise to 700 million by 2045.^[1]^ The impact of DM and its complications extends beyond individuals and families, imposing substantial economic burdens and social instability on countries across the globe.^[2]^ Research indicates that around one-third of patients with diabetes also suffer from cardiovascular disease (CVD), with a risk of developing CVD that is 2 to 4 times higher than that of individuals without diabetes. Moreover, the risk of hospitalization due to heart failure (HF) is also doubled in diabetic patients.^[3,4]^ DM exacerbates CVD through hyperglycemia-induced endothelial dysfunction, oxidative stress, and chronic inflammation, accelerating atherosclerosis.^[3,5]^ Globally, diabetes-related CVD deaths increased by 41% from 2005 to 2018, with heightened vulnerability in low-income populations.^[5]^ Evidence has shown a positive correlation between CVD risk in patients with DM and levels of glycated hemoglobin (HbA1c), with over 70% of individuals with type 2 DM (T2DM) exhibiting hypertension or dyslipidemia.^[5,6]^ While strict glycemic control has shown limited efficacy in reducing the incidence and mortality risk of CVD in patients with T2DM, comprehensive interventions targeting multiple risk factors have demonstrated a more significant impact in reducing both CVD occurrence and mortality rates.^[7]^ Ethnic minorities (such as non-Hispanic Blacks), low-income individuals (PIR ≤ 1.3), and elderly populations experience disproportionate DM-CVD burdens due to healthcare disparities and dietary inequities.^[8,9]^ However, further investigation is needed to clarify the exact relationship between lifestyle factors, including dietary habits and weight management, and the development of CVD in individuals with DM.

Recent studies have increasingly revealed the inflammatory mechanisms associated with DM.^[10]^ DM-associated inflammation involves adipose tissue macrophage infiltration, NOD-like receptor family, pyrin domain containing 3 inflammasome activation, and interleukin-1β (IL-1β)/interleukin-6-driven insulin resistance.^[8,11]^ Hyperglycemia also primes neutrophil extracellular trap formation, exacerbating vascular damage. Dietary management, a key component in the treatment and management of DM, not only focuses on glycemic control but also involves the relationship between dietary habits and inflammation, which plays a significant role in the incidence of diabetes and its complications.^[12,13]^ Similarly, dietary management is equally important in the context of CVD.^[14]^ The dietary inflammatory index (DII) has emerged as a promising new measure that reflects the inflammatory potential of dietary habits.^[15]^ The DII is derived from dietary data encompassing 1943 studies across 11 countries, summarizing the correlation between dietary components and 6 inflammatory cytokines, including IL-1β. This dietary assessment tool integrates 36 anti-inflammatory components and 9 pro-inflammatory components, calculated using the formula: *Z* score = (A daily intake − A global mean daily intake)/standard deviation of A global mean daily intake × A inflammatory effect score, where A represents the dietary component. After transforming *Z* into a percentile and adjusting to a symmetric distribution centered around “0,” the overall inflammatory score for each dietary component is determined and summed to obtain an individual’s DII.^[16]^ As a new quantitative measure for evaluating dietary inflammatory potential, the DII has been widely applied in various chronic disease studies, particularly in relation to DM.^[17,18]^ The DII quantifies diet’s inflammatory potential using 45 dietary components linked to 6 inflammatory biomarkers.^[14]^ A higher DII predicts gestational diabetes (odds ratio [OR] = 1.23; 95% confidence interval [CI]: 1.09–1.38), pediatric fatty liver disease (β = 0.34, *P* < .001), and obesity phenotypes (OR = 1.15; 95% CI: 1.02–1.30),^[18–20]^ underscoring its clinical relevance. DII quantifies diet’s inflammatory impact, independently predicting T2DM incidence and CVD mortality in meta-analyses.^[15,19]^ Its application in diabetic cohorts offers actionable insights for dietary interventions. However, there remains a lack of clinical evidence regarding the DII in patients with comorbid DM and CVD.

Additionally, the systemic immune-inflammation index (SII) has recently been applied as a valuable indicator for assessing prognosis and survival curves in patients with CVD.^[21]^ The SII is a novel biomarker that provides a more comprehensive evaluation of immune status and inflammation.^[22]^ Complete blood counts are not only frequently performed in laboratory tests in clinical settings but also serve as important indicators of systemic inflammation and/or immune suppression, proving beneficial in the context of CVDs.^[23]^ Consequently, the SII emerges as a highly convenient, efficient, and comprehensive biomarker. The SII has been associated with CVDs and has been observed to have a negative correlation with clinical outcomes in individuals suffering from acute coronary syndrome, HF, ischemic stroke, and hypertension.^[24,25]^ Moreover, recent studies have begun to explore the potential mechanisms of SII in the interplay between DM and CVD.^[25,26]^ SII integrates platelets (thrombosis), neutrophils (tissue damage), and lymphocytes (immune dysregulation), reflecting systemic inflammation that accelerates atherosclerosis in DM.^[21,24,25]^ However, current research primarily focuses on the ability of the SII to more accurately predict major cardiovascular events and mortality, alongside its correlation with the severity of coronary artery disease. While inflammation links diet to CVD in DM,^[13,16]^ clinical evidence on the mediating role of SII remains sparse.

Thus, this study aims to analyze the association between DII and CVD in individuals with DM while further investigating the role of the SII and other related factors in this context. By elucidating these interactions, we seek to explore potential mediating effects and influences that may exist in the comorbidity of DM and CVD, contributing to a better understanding of the underlying mechanisms involved. This is the first study to validate SII and body mass index (BMI) as dual mediators between DII and CVD in a nationally representative diabetic cohort, offering translational strategies for risk stratification.

## 2. Methods

### 2.1. Study population

**Figure 1.**
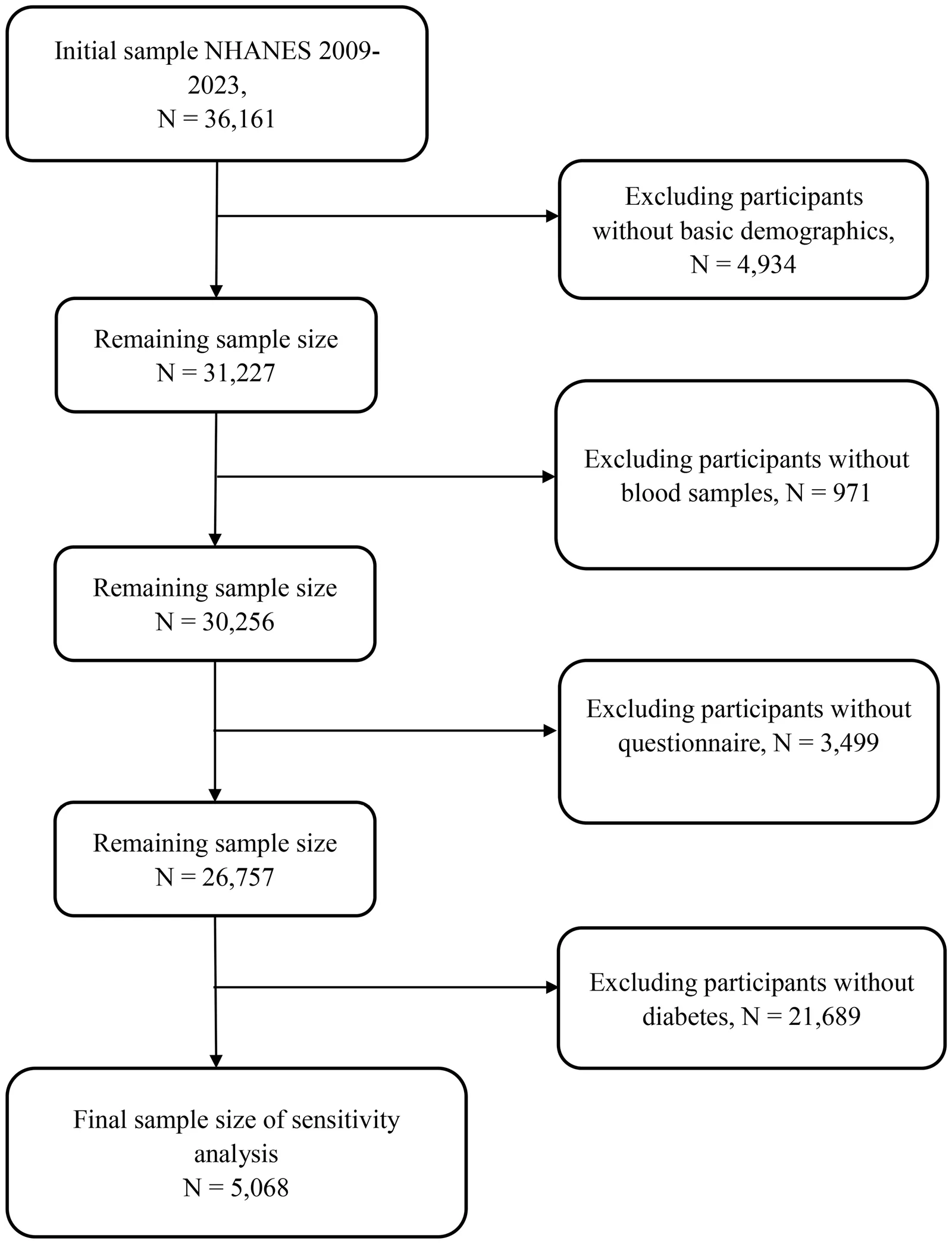
Flowchart for screening of research subjects. Sensitivity analysis in 5068 participants confirmed robust mediation effects. NHANES = National Health and Nutrition Examination Survey.

The National Health and Nutrition Examination Survey (NHANES) serves as a comprehensive, cross-sectional, population-based study designed to assess the lifestyle, dietary habits, and health status of the noninstitutionalized civilian population in the United States. This analysis utilized NHANES data from the periods 2009 to 2010, 2011 to 2012, 2013 to 2014, 2015 to 2016, 2017 to 2020, and 2021 to 2023. Detailed information regarding the study design and data collection methodologies is available on the National Center for Health Statistics website.^[27,28]^ Participants are not followed longitudinally across survey years, minimizing retesting bias in our analysis.^[28]^ The inclusion criteria were adults (≥20 years) with DM (diagnosis/HbA1c ≥6.5%/medication use). The exclusion criteria were pregnancy, cancer, incomplete DII/SII data, or institutionalized individuals. A total of 36,161 participants aged 20 years or older were initially identified, from which 5068 participants with complete data were included in the final analysis. The screening process is illustrated in Figure 1.

### 2.2. Assessment of DM and CVD

DM was defined using one of the following criteria: a previous diagnosis by a medical professional, fasting plasma glucose levels of ≥7.0 mmol/L, HbA1c levels of ≥6.5 mmol/L, or the use of diabetes medications.^[29]^

To assess the presence of CVD, participants were asked, “Has a doctor or other health expert ever informed you that you have CHF/CHD/angina pectoris/MI/stroke?” Individuals responding “yes” to any of these conditions were classified as having CVD.^[30]^

### 2.3. Assessment of DII

In NHANES, dietary intake data were gathered through two 24-hour dietary recall interviews. The DII was calculated using a modified version of the previously developed method.^[15,31]^ This approach incorporates data on 26 nutrients, including alcohol, vitamins A, B6, B12, C, D, and E, caffeine, carbohydrates, cholesterol, total fat, fiber, Fe, Mg, Zn, Se, monounsaturated fatty, polyunsaturated fatty acid, niacin, n − 3 fatty acids, n − 6 fatty acids, protein, riboflavin, saturated fat, thiamin, and β-carotene. Notably, DII scores were obtainable even when the nutrients used for the DII calculation were fewer than 30.^[31]^

### 2.4. Assessment of SII

In NHANES, laboratory examinations, including routine blood tests, were conducted within 24 hours of admission. SII was calculated using neutrophil, platelet, and lymphocyte counts from routine blood tests via the formula^[32]^:


$$SII=\frac{neutrophil\;count\times platelet\;count}{lymphocyte\;count}$$

### 2.5. Covariates

The following variables were included as covariates due to their potential confounding effects: age, sex, ethnicity (non-Hispanic White, non-Hispanic Black, and Others), education level (less than high school, high school or equivalent, college or above), household income, and BMI, all of which were obtained through self-report. Household income, measured by the poverty-income ratio (PIR), was categorized into 3 levels: high (>3.5), middle (1.3–3.5), and low (≤1.3).^[33]^ BMI was categorized as follows: normal or low weight (<25.0 kg/m^2^), overweight (25.0–29.9 kg/m^2^), or obese (≥30 kg/m^2^).^[34]^

### 2.6. Data analysis

All the data were consolidated into a single dataset following the NHANES protocol. The data analyses considered the masked variance and used the recommended weighting methodology. A generalized linear model was employed to examine the influence of the DII on CVD among individuals with DM. The results were reported as ORs along with 95% CIs and *P* values. Models were adjusted for major sociodemographic and clinical confounders. Additionally, a mediation effect model was utilized to explore the relationships between DII, BMI, SII, and CVD in diabetes. The mediation effect analysis was conducted using Statistical Package for the Social Sciences (PROCESS Macro Model 4; SPSS 26.0 software; IBM Corp.), while all other analyses were performed using statistical software R (version 4.2.2; R Foundation for Statistical Computing, https://www.r-project.org/). A 2-sided *P* value of <0.05 was considered statistically significant.

## 3. Results

### 3.1. Demographics

Table 1 shows the characteristics of CVD among participants with DM from NHANES 2009 to 2023. Among 5068 participants, 1243 (24.53%) had DM with CVD. Age, sex, race, education level, PIR, BMI, DII, neutrophil, lymphocyte, monocyte, platelet, and SII differed between participants with DM with CVD and those without CVD. Underweight (BMI <18.5 kg/m^2^) was combined with normal weight due to the small sample size (n = 45) and similar metabolic profiles.

**Table 1:** Characteristics of the NHANES 2009 to 2023 participants with diabetes.

Characteristics	Total (N = 5068)	Without CVD (N = 3825, 75.47%)	CVD (N = 1243, 24.53%)	*P* value
Age, mean ± SD*	60.21 ± 13.67	58.00 ± 13.82	67.03 ± 10.61	<.001§
Sex, n (%)†				<.001§
Male	2654 (52.37)	1920 (50.20)	734 (59.05)	
Female	2414 (47.63)	1905 (49.80)	509 (40.95)	
Race, n (%)†				<.001§
Non-Hispanic White	1877 (37.04)	1290 (33.72)	587 (47.22)	
Non-Hispanic Black	1223 (24.13)	934 (24.42)	289 (23.25)	
Other	1968 (38.83)	1601 (41.86)	367 (29.53)	
Education level, n (%)†				<.001§
Less than high school	1418 (27.98)	1033 (27.01)	385 (30.97)	
High school or equivalent	2720 (53.67)	2036 (53.23)	684 (55.03)	
Above high school	930 (18.35)	756 (19.76)	174 (14.00)	
PIR, mean ± SD‡	2.34 ± 1.56	2.40 ± 1.58	2.16 ± 1.49	<.001§
PIR, n (%)†				<.001§
Low (≤1.3)	1745 (34.43)	1253 (32.76)	492 (39.58)	
Middle (1.3–3.5)	2024 (39.94)	1537 (40.18)	487 (39.18)	
High (>3.5)	1299 (25.63)	1035 (27.06)	264 (21.24)	
BMI, mean ± SD, kg/m^2^*	32.66 ± 7.80	32.51 ± 7.75	33.11 ± 7.92	.018§
BMI, n (%)†				.043§
Normal or underweight	669 (13.20)	529 (13.83)	140 (11.26)	
Overweight	1419 (28.00)	1076 (28.13)	343 (27.60)	
Obesity	2980 (58.80)	2220 (58.04)	760 (61.14)	
DII, mean ± SD‡	1.49 ± 1.69	1.52 ± 1.69	1.89 ± 1.67	<.001§
Neutrophil, mean ± SD, 10^9^/L‡	4.61 ± 1.95	4.56 ± 1.98	4.73 ± 1.83	.001§
Lymphocyte, mean ± SD, 10^9^/L‡	2.17 ± 0.87	2.22 ± 0.81	2.03 ± 1.01	<.001§
Monocyte, mean ± SD, 10^9^/L‡	0.58 ± 0.22	0.57 ± 0.22	0.61 ± 0.23	<.001§
Platelet, mean ± SD, 10^9^/L‡	237.22 ± 70.22	242.21 ± 69.84	221.85 ± 69.19	<.001§
SII, mean ± SD, 10^9^/mmol‡	570.71 ± 417.88	559.51 ± 392.32	605.20 ± 486.76	.002§

BMI = body mass index, CVD = cardiovascular disease, DII = dietary inflammatory index, NHANES = National Health and Nutrition Examination Survey, PIR = poverty-income ratio, SD = standard deviation, SII = systemic immune-inflammation index.

* One-way ANOVA.

† Chi-square test.

‡ Mann–Whitney *U* test.

§ Significant correlation, *P* < .05.

### 3.2. Effects of DII on CVD

The results in Table S1, Supplemental Digital Content 1, describe the relationship between DII and CVD. Before adjusting for confounders, OR = 1.142 (95% CI: 1.098–1.188, *P* < .001). The adjusted model (age, sex, race, education, income, and BMI) showed an OR = 1.140 (95% CI: 1.091–1.192, *P* < .001). This confirms DII as a reliable clinical risk marker.

### 3.3. DII, BMI, SII, and CVD among adults with DM

**Figure 2.**
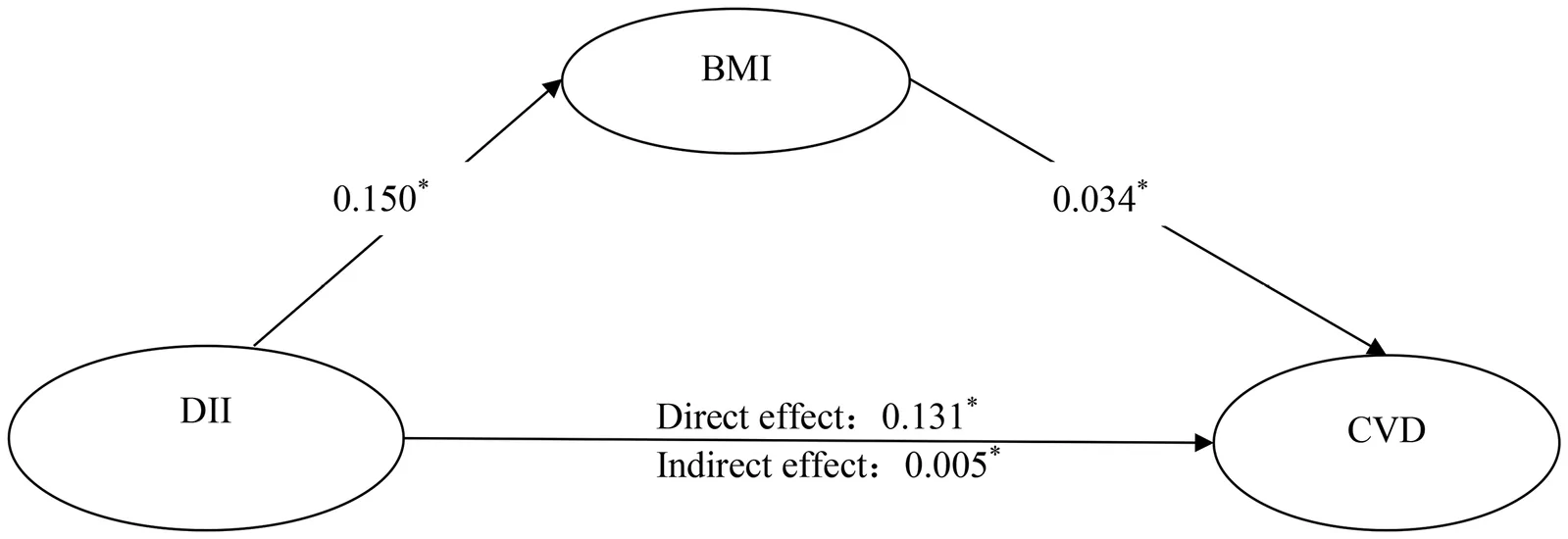
The mediating effect of BMI in the relationship between DII and CVD among adults with diabetes. *: *P* < .05. BMI = body mass index, CVD = cardiovascular disease, DII = dietary inflammatory index.

**Figure 3.**
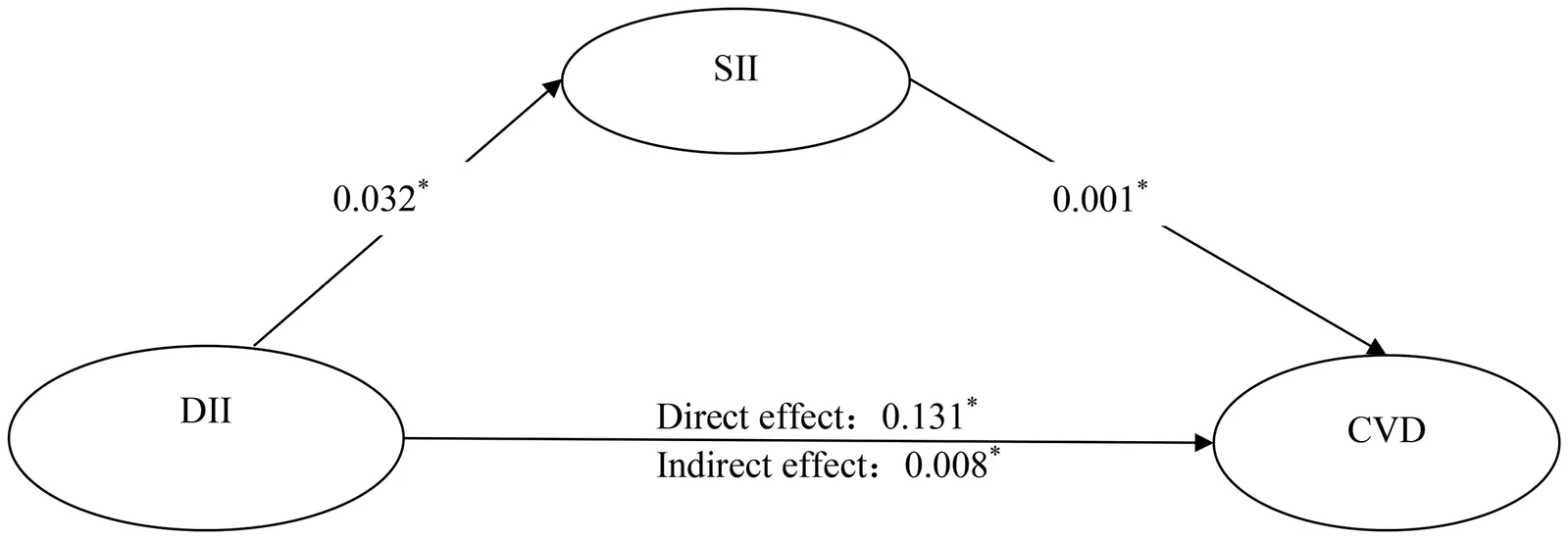
The mediating effect of SII in the relationship between DII and CVD among adults with diabetes. *: *P* < .05. CVD = cardiovascular disease, DII = dietary inflammatory index, SII = systemic immune-inflammation index.

Table S2, Supplemental Digital Content 2, presents a comprehensive correlation matrix among diabetic patients, revealing significant interrelationships between key variables. DII demonstrates strong positive correlations with male sex (*r* = 0.236, *P* < .01) and BMI (*r* = 0.063, *P* < .01), while showing significant negative correlations with education level (*r* = −0.176, *P* < .01) and income-to-poverty ratio (PIR; *r* = −0.198, *P* < .01). CVD exhibits significant positive associations with age (*r* = 0.290, *P* < .01), DII (*r* = 0.094, *P* < .01), SII (*r* = 0.044, *P* < .01), and BMI (*r* = 0.033, *P* < .05). Notably, CVD risk is inversely related to socioeconomic factors, including PIR (*r* = −0.067, *P* < .01), education (*r* = −0.061, *P* < .01), and race (non-White status; *r* = −0.128, *P* < .01). These interconnected patterns highlight that pro-inflammatory diets (higher DII) cluster with male sex, lower socioeconomic status, and elevated BMI, collectively contributing to increased CVD risk in diabetes through inflammatory pathways. Table S3, Supplemental Digital Content 3, demonstrates the direct/indirect associations of DII, BMI, SII, and CVD adjusted for demographic and socioeconomic characteristics (age, sex, race, education level, and PIR). BMI partially mediated the relationship between DII and CVD among adults with DM, with an effect coefficient of 0.005 (95% CI: 0.001–0.010), indicating that BMI explained 3.82% of the effect of the DII on CVD (Fig. 2). SII partially mediated the relationship between DII and CVD among adults with DM, with an effect coefficient of 0.008 (95% CI: 0.001–0.016), indicating that SII explained 6.11% of the effect of DII on CVD (Fig. 3).

## 4. Discussion

This study is the first to report that DII is a significant factor associated with the occurrence of CVD in diabetic patients. It underscores the critical importance of dietary management, particularly anti-inflammatory diets, in preventing CVD among individuals with DM. Furthermore, our findings emphasize the need for effective control of BMI alongside dietary management. Specifically, we demonstrate that a combination of weight reduction and dietary intervention significantly reduces the incidence of CVD. Notably, this research also highlights the role of SII as an important indicator for the onset of CVD, extending its utility beyond merely predicting mortality rates.

In contrast to previous studies, our results indicate elevated CVD risk in males with DM compared with females, regardless of diabetes type. Earlier research identified standardized mortality rates for CVD among individuals with type 1 diabetes mellitus acid as 8.8 for men and 24.7 for women.^[35]^ A report on the incidence of CVD in European populations with type 1 diabetes mellitus acid indicated a prevalence of 9% in men and 10% in women, with rates increasing with age.^[36]^ These findings differ from our results, which indicate a greater susceptibility to CVD among male patients with DM. This discrepancy may be attributed to variations in diabetes types and demographic factors, including ethnicity and sex differences.^[37]^ Additionally, our study observed significant statistical differences based on ethnicity and living environments, further supporting this hypothesis. Non-Hispanic Whites showed higher CVD prevalence (47.2% vs 23%–30% in Others), potentially reflecting healthcare access disparities. Rural residents had 1.3× higher CVD risk (*P* = .02), suggesting environmental influences on diet and inflammation.

One of the key conclusions of this study is the demonstrable relationship between the DII and the incidence of CVD in individuals with DM. DII serves as a novel tool to investigate the inflammatory pathogenic potential of various dietary components, specifically quantifying the inflammatory potential of diets.^[38]^ A pro-inflammatory diet is characterized by a higher DII score, while an anti-inflammatory diet is represented by a lower score. The DII scores are standardized based on global dietary intake patterns, accommodating diverse cultural eating habits.^[38]^ Research indicates a direct correlation between specific dietary components and inflammation. Focusing on overall dietary quality, rather than isolating individual foods, offers a more accurate understanding of the mechanisms underlying inflammation.^[11]^ Studies link anti-inflammatory diets to a reduced risk of modifiable chronic diseases such as obesity, T2DM, and CVD.^[39]^ Potential mechanisms underlying inflammation may include genomic instability, cellular senescence, dysregulation of immune cells, mitochondrial dysfunction, changes in microbial composition, and chronic infections.^[40]^ Elevated dietary inflammatory risk may enhance the levels of inflammatory markers such as interleukin-4, interleukin-6, interleukin-10, and IL-1β, while also accelerating telomere shortening. Typically, pro-inflammatory diets are associated with a higher intake of saturated fats, which significantly correlate with increased risks of CVD occurrence and mortality.^[41]^ Furthermore, a study conducted in the United States demonstrated that individuals consuming pro-inflammatory diets faced a 38% higher risk of developing CVD, reinforcing our assertion that DII is a crucial factor in the relationship between DM patients and the risk of CVD.^[42]^

Interestingly, our research is the first to examine patients with DM who also have a high DII as an observed population to explore the mediating effects of CVD in this population. We found that BMI and SII serve as significant mediators, indicating an elevated risk of CVD in these patients. Previous studies have established links between high-DII diets and conditions such as metabolic syndrome, hypertension, hyperglycemia, abdominal obesity, and hypertriglyceridemia. An increase in DII correlates with a higher incidence of abdominal obesity and hyperglycemia in overweight individuals.^[43]^ Additionally, higher DII scores are associated with increases in BMI, weight gain, and obesity, all of which are related to chronic systemic and localized low-grade inflammation.^[16]^ A study focusing on men has confirmed a significant rise in DII scores with advancing age. Furthermore, research has shown a positive correlation between DII and both age and obesity indices, suggesting that diet may play a role in the onset of obesity in older adults through inflammatory pathways.^[44]^ In a cohort study involving individuals aged 20 to 90, pro-inflammatory diets, measured by high DII scores, were associated with increased CVD mortality in individuals with central obesity, even within a normal BMI range.^[45]^ However, we are the first to propose the mediating role of BMI within this comorbid population, suggesting that weight management may be an effective strategy for reducing the risk of CVD in patients with DM and high-DII dietary patterns. A prior Mendelian randomization study investigating the causal relationship between BMI, inflammatory indices, and T2DM revealed that DII is not a cause of T2DM or obesity; instead, chronic inflammation may be a common outcome of both.^[46]^ This hypothesis validates our earlier assertions. We propose that patients with DM who follow high-DII diets may experience exacerbated chronic inflammation under the risk factors of high BMI and obesity, ultimately leading to increased incidence and mortality of CVD.^[46]^ Therefore, while adopting a low-DII diet may help manage some aspects of chronic inflammation, it is crucial not to overlook or conflate the direct effects and influences of DM and obesity on chronic inflammation.

Furthermore, this study identifies the significant mediating role of SII in the occurrence of CVD among patients with DM and high DII.^[25]^ Previous research has linked high-risk cardiovascular conditions, such as atherosclerosis, HF, and apoptosis-speck-like protein containing a CARD, to persistently high levels of inflammation. In DM patients, peripheral arterial disease often affects medium-sized arteries such as the profunda femoris and anterior tibial arteries, with atherosclerosis being the primary cause. This condition shares common pathological mechanisms, such as endothelial dysfunction and oxidative stress, with other thrombotic cardiovascular disorders, including coronary artery disease and cerebrovascular disease.^[3]^ These insights underscore the critical role of inflammatory mechanisms in the pathogenesis of CVD among DM patients. SII, which integrates the counts of platelets, lymphocytes, and neutrophils in peripheral blood, effectively assesses the body’s systemic inflammatory and immune status. Compared with isolated blood cell counts, SII demonstrates greater stability, as it is less susceptible to fluctuations caused by dehydration and fluid overload. A study by Chen et al highlighted SII as a valuable predictive biomarker for diabetes.^[47]^ Our research emphasizes the importance of utilizing SII to assess and identify the risk of CVD in the high-DII DM population, providing a new reference for early prediction and intervention strategies in the comprehensive management of DM and CVD. It is essential to note that our study population is inherently composed of DM patients with high DII scores. We propose that chronic inflammation induced by high DII can activate endothelial cells and trigger the release of inflammatory mediators, ultimately impairing endothelial function. Additionally, DII may further influence SII, as endothelial dysfunction is commonly regarded as a precursor to atherosclerosis and various CVDs. Notably, SII is often recognized as a vital indicator of CVD mortality. For instance, research conducted by Long et al revealed a nonlinear relationship between SII levels and CVD mortality.^[21]^ Their findings suggest that low SII levels may indicate reduced neutrophil or platelet counts, which are frequently observed in populations with poor prognosis and critical health conditions. This does not conflict with our study, as our cohort comprises DM patients with elevated DII, focusing on the mediating role of SII in the occurrence of CVD in this demographic. It is worth discussing that the U-shaped correlation observed with lower SII values may correspond to disease severity and mortality. Therefore, maintaining an appropriate SII is crucial, as extreme elevations or significant reductions can have adverse health implications.

This study presents several limitations. First, we explored potential correlating factors between CVD and DM in individuals with a high DII, proposing that weight management and monitoring specific indicators may reduce the incidence and mortality of CVD. However, as a cross-sectional study with a relatively small sample size, our findings necessitate further case-control and prospective studies to elucidate the relationship between DII and cardiovascular metabolic diseases, as well as their connections to nutritional status comprehensively. Additionally, the data were primarily derived from questionnaire responses. Subsequent research in real-world cohorts is essential to further investigate the role of inflammatory mechanisms in the co-occurrence of DM and CVD. Furthermore, our findings suggest that SII serves as a significant mediator, indicating a need for future studies to incorporate more specific metrics to quantify the direct inflammatory relationships and mechanisms between these entities.

## 5. Conclusion

Among adults with diabetes, a higher DII was independently associated with an increased prevalence of CVD. BMI and SII partially mediated this relationship, suggesting that adiposity and chronic inflammation may represent important mechanistic pathways. These findings highlight the potential value of dietary inflammation assessment, weight management, and inflammatory risk stratification in identifying individuals with diabetes at higher cardiovascular risk.

## Acknowledgments

The authors thank the National Center for Health Statistics staff for their assistance in explaining and accessing the NHANES variables and thank the NHANES participants and the data collection team for making this survey possible.

## Author contributions

**Conceptualization:** Shaofang Tang, Hongtao Li, Zixun Wang, Zhiqing Li, Yin Li, Chenlin Dai.

**Formal analysis:** Shaofang Tang, Hongtao Li, Zixun Wang, Baoping Wang.

**Funding acquisition:** Zixun Wang.

**Project administration:** Zhiqing Li, Yin Li, Chenlin Dai.

**Supervision:** Zhiqing Li, Yin Li, Chenlin Dai.

**Writing – original draft:** Shaofang Tang, Hongtao Li, Zixun Wang, Baoping Wang.

**Writing – review & editing:** Zhiqing Li, Yin Li, Chenlin Dai.






