# Supplementary material for: Mediating roles of systemic immune-inflammation index and body mass index in the association between dietary inflammatory index and cardiovascular disease in diabetes
Source: Medicine (Baltimore). 2026 Sep 11;105(37):e50622. doi: 10.1097/MD.0000000000050622 (PMC13574442; doi:10.1097/MD.0000000000050622)
Supplement: Supplementary file 1 [file medi-105-e50622-s001.docx]

Table S1 Association between DII and CVD among diabetes.

| Variables | Crude model | | Adjusted model | |
| --- | --- | --- | --- | --- |
|  | OR (95%CI) | *p* value | OR (95%CI) | *p* value |
| CVD |  |  |  |  |
| No | Ref | Ref | Ref | Ref |
| Yes | 1.142 (1.098, 1.188) | <0.001^*^ | 1.140 (1.091, 1.192) | <0.001^*^ |

Notes: Models adjusted for age, sex, race, educational level, PIR, and BMI.

CI, confidence interval; PIR, poverty income ratio; BMI, body mass index; DII, dietary inflammatory index; CVD, Cardiovascular Disease;.

*Significant correlation, P<0.05.
