# Supplementary material for: Mediating roles of systemic immune-inflammation index and body mass index in the association between dietary inflammatory index and cardiovascular disease in diabetes
Source: Medicine (Baltimore). 2026 Sep 11;105(37):e50622. doi: 10.1097/MD.0000000000050622 (PMC13574442; doi:10.1097/MD.0000000000050622)
Supplement: Supplementary file 2 [file medi-105-e50622-s002.docx]

Table S2 Correlation matrix between variables among diabetes.

| Variables | 1 | 2 | 3 | 4 | 5 | 6 | 7 | 8 | 9 |
| --- | --- | --- | --- | --- | --- | --- | --- | --- | --- |
| 1. Age | − |  |  |  |  |  |  |  |  |
| 2. Sex | −0.051^**^ | − |  |  |  |  |  |  |  |
| 3. Race | −0.190^**^ | 0.018 | − |  |  |  |  |  |  |
| 4. Education level | −0.049^**^ | −0.053^**^ | −0.174^**^ | − |  |  |  |  |  |
| 5. PIR | 0.048^**^ | −0.124^**^ | −0.141^**^ | 0.434^**^ | − |  |  |  |  |
| 6. BMI | −0.200^**^ | 0.152^**^ | −0.104^**^ | −0.010 | −0.034^**^ | − |  |  |  |
| 7. SII | −0.001 | 0.064^**^ | −0.108^**^ | 0.056^**^ | 0.013 | 0.088^**^ | − |  |  |
| 8. DII | 0.035^*^ | 0.236^**^ | 0.002 | −0.176^**^ | −0.198^**^ | 0.063^**^ | 0.029^*^ | − |  |
| 9. CVD | 0.290^**^ | −0.076^**^ | −0.128^**^ | −0.061^**^ | −0.067^**^ | 0.033^*^ | 0.044^**^ | 0.094^**^ | − |

Notes: *Significant correlation, P<0.05; **, P<0.01.

PIR, poverty income ratio; BMI, body mass index; DII, dietary inflammatory index; SII, systemic immune inflammation index; CVD, Cardiovascular Disease.
