# Supplementary material for: Mediating roles of systemic immune-inflammation index and body mass index in the association between dietary inflammatory index and cardiovascular disease in diabetes
Source: Medicine (Baltimore). 2026 Sep 11;105(37):e50622. doi: 10.1097/MD.0000000000050622 (PMC13574442; doi:10.1097/MD.0000000000050622)
Supplement: Supplementary file 3 [file medi-105-e50622-s003.docx]

Table S3 The direct and indirect effects of DII and BMI on CVD among diabetes.

| Variables | β | Boot SE | Boot CI | |
| --- | --- | --- | --- | --- |
|  |  |  | Lower Limit | Upper Limit |
| BMI |  |  |  |  |
| Direct effect | **0.131^*^** | 0.023 | 0.087 | 0.175 |
| Indirect effect | **0.005^*^** | 0.002 | 0.001 | 0.010 |
| SII |  |  |  |  |
| Direct effect | **0.131^*^** | 0.020 | 0.092 | 0.171 |
| Indirect effect | **0.008^*^** | 0.003 | 0.001 | 0.016 |

Notes: *: P<0.05; BMI, body mass index; DII, dietary inflammatory index; SII, systemic immune inflammation index; CVD, Cardiovascular Disease; Boot SE, bootstrap standard error; CI, Confidence Interval; Boot CI, bootstrap CI.

All models were adjusted for age, sex, race, education level and PIR.
